# Exposure of hairdressing apprentices to airborne hazardous substances

**DOI:** 10.1186/1476-069X-5-23

**Published:** 2006-08-07

**Authors:** Estelle Mounier-Geyssant, Véronique Oury, Lory Mouchot, Christophe Paris, Denis Zmirou-Navier

**Affiliations:** 1 INSERM Research unit ERI n°11, Vandoeuvre-les-Nancy, France; 2 INRS, Department of exposure assessment, Vandoeuvre-les-Nancy, France; 3 School of Medicine, Nancy University; France

## Abstract

**Background:**

Few studies have investigated exposure of hairdressing apprentices to airborne irritants. This study describes exposure levels of apprentices to chemical products used in hairdressing salons in relation with their activity.

**Methods:**

Following a two stages study design, a group of 300 students completed a questionnaire on their work activities and environment. Among these, a group of 28 subjects volunteered to undergo personal exposure and workplace concentrations measurements over a work shift, during a cold and a hot season, with the agreement of the salon owners. Three chemical substances were studied (ammonia, hydrogen peroxide and persulfates) because they are respiratory tract irritants and because their concentrations could be quantified within a 5 to 8 hour shift period.

**Results:**

Personal exposure values for H_2_O_2_and NH_3_ (averages [standard deviations] are 0.05 [0.04] and 0.90 [0.76] mg.m^-3^, respectively) were greater than workplace ambient air concentrations (corresponding values of 0.04 [0.03] and 0.68 [0.42] mg.m^-3^) for H_2_O_2 _and NH_3_, with no significant seasonal variation. By contrast, workplace concentrations of persulfates (0.019 [0.018] mg.m^-3^) were greater than personal exposure (0.016 [0.021] mg.m^-3^, a finding that is consistent with the fact that bleaching is more often undertaken by senior hairdressers. However, all exposure values were lower than the current TLV TWA values. This study also shows that over half of technical spaces where chemical substances used for dying, permanenting or bleaching are manipulated, have no ventilation system, and not even a door or a window opening outside.

**Conclusion:**

The study hairdressing salons, on average, were small, the most probable reason why occupational hygiene measures such as appropriate ventilation were too seldom implemented. As a consequence, young apprentices and senior hairdressers experience substantial exposure to known airways irritants.

## Background

Occupational asthma is caused by sensitisation to an agent inhaled in the workplace. Onset of symptoms occurs after repeated exposure to chemicals and allergens in the work environment [1,2]. However, while many subjects experience exposure to agents that are known or suspected to provoke occupational asthma, only a fraction develop the condition [3,4]. Hairdressers are exposed to a variety of chemical agents as a result of usage of several hair cosmetic products such as hair dyes, permanent wave solutions and bleaches. Several constituents of hair care products are airway irritants and may induce respiratory conditions, including impairment of the pulmonary function and chronic bronchitis [3,5-12]. Hairdressing is described as an occupation at risk of asthma [4,13-17] and exposure to persulfate salts is involved in the induction of occupational asthma [3,16,18,19].

Exposure studies in hairdressing salons are few. Given the variety of chemical substances that are used, some authors have measured tracer agents such as hydrogen peroxide, ammonia, volatile organic compounds (toluene, ethanol, isopropanol, ether, diaminotoluene, phenylenediamine) and carbon dioxide [20-23]. A 1992 study showed that workplace concentrations of phenylenediamine were below the ACGIH TLV-TWA [20]. Ethanol was used to categorize hairdressing workplace concentrations according to ventilation levels and intensity of technical services [21]. CO_2 _concentrations and levels for ammonia and ethanol were found high in absence of mechanical ventilation [22]. Installation of ventilation was shown to reduce measured levels of ethanol, isopropanol, toluene and ammonia [23].

Hairdressing apprentices experience exposure to the same substances during their training activities. Health studies among apprentices are sparse, however. Variability of the peak expiratory flow was assessed in Florence, Italy, across 10 days and was associated with job tasks, with influences of gender and smoking habits [24]. The prevalence of occupational asthma was 1.7 % among 116 apprentices [25]. Hairdressing apprentices exhibited poorer lung function values than office apprentices [26]. In the framework of a study that aimed to assess how non asthmatic apprentices may develop airway inflammation in the course of their 3 years training, we undertook an exposure study to a selected number of airways irritants that are emitted into the air when using hairdressing products. We measured personal exposures and workplace concentrations during a work shift, with repeated sampling across two (hot and cold) seasons. This paper also describes the tasks that involve contact with such products during hairdressing activities.

## Methods

With the collaboration of the administration of a large apprenticeship school in Nancy, north-east of France, we conducted a cross-sectional study in different hairdressing classes during the 2003–2004 and 2004–2005 academic years. This study had two steps. First, all apprentices following the first, second or third year of their training programme during this period were asked to complete, in the classroom, a questionnaire describing their work environment and activities. In a second step, volunteers were asked to participate to exposure measurements while at work in their hairdressing salon. The training programme typically encompasses a period of 3 weeks of practical work in the salon, followed by one week at school for practical and academic classes. Except for specific reasons (such as being fired by the hairdresser or because of resignation) an apprentice works in the same salon until he/she graduates. Measurements were done during a regular workday, most of the time between Monday and Friday, depending upon agreement of the salon owners. With a view to assess seasonal variations, personal and workplace measurements were repeated over two seasons.

### Completion of questionnaires

Questionnaire was designed after review of the literature, observations in several hairdressing salons and visits during the practical training classes in the apprenticeship school. It was validated by the school instructors and the heads of the local and regional hairdressing federations. A pilot study was undertaken during academic year 2002–2003 to check suitability of the questions for the study population.

In addition to retrieving information on personal characteristics, duration of a work shift, spatial organization of the salon and ventilation characteristics, the questionnaire requested the students to describe in detail their activities during a "standard" workday in the salon, "standard" meaning the most recent day with about the average daily number of customers over the last month.

### Exposure measurements in salons

Because apprentices have little technical activities during the first year of training, and for schedule availability reasons, volunteers were mostly recruited among second graders. After apprentices declared their willingness to participate in this second stage of the study, authorization to undertake the measurement study was requested from the corresponding hairdressing salon owners or managers. Before returning a signed consent form, all study subjects were given a letter of invitation explaining the aim of the study and providing information on occupational asthma and its prevention.

This exposure study involved personal measurements and fixed site monitoring in different salon locations: (i) in the technical space of the customer area (where customers sit during and after applying permanent waving, hair colour or bleaches), (ii) near the hair wash area and (iii) in the 'technical room' (i.e. where chemical mixtures are prepared, a location that is often in a rear part of the salon or in a specific room). Number of sampling locations depended upon the salon spatial characteristics. During air sampling, we collected information on relevant salons' characteristics (salon volume; existence of mechanical ventilation; means of natural ventilation – i.e. number of doors or windows opening directly outside the day measurements were done).

With a few exceptions, all apprentices contributed to two series of personal exposure and fixed site measurements: during the "cold season", from February to April, and during the "hot" season", from April to August. During the measurements, information was retrieved on usage of products that contained the selected study compounds. Among the variety of chemicals used in the hairdressing process, three compounds were chosen after review of the literature: ammonia (NH_3_), hydrogen peroxide (H_2_O_2_) and persulfates (H_2_S_2_0_8_). Hair dyeing formulations belong to three categories [23,27] used for temporary, semi-permanent and permanent hair colouring. Colours of temporary dyes are readily removable with a single shampoo. The colouring effects of the semi-permanent dyes gradually fade over a period of 4 to 6 weeks, following the levels of hydrogen peroxide, ammonia and artificial colour molecules that have been applied. Permanent hair dyes, also called oxidative hair dyes, contain adjusting agents such ammonia and a stabilised solution of hydrogen peroxide. Oxidative hair dyes are resistant to fading by shampooing [23,27]. Hair bleaches contain ammonium, sodium and potassium persulfates. The active ingredients are persulfates which are mixed with an oxidant (hydrogen peroxide) just before usage. In order to improve the hair penetration, ammonia releasers such as ammonium chloride or ammonium phosphate are added. Permanent waving chemicals are of two types: alkaline or slightly acidic water solutions. They contain salts of thioglycolic acid and hydrogen peroxide. Like for hair bleaching, ammonia is added to enhance hair penetration [23,27]. As a result, ammonia and hydrogen peroxide are ingredients of permanent waves, hair dyes and hair bleaching. Persulfates are used in the formulation of bleaching powders. Selection of these three compounds was also decided after a feasibility study, both in one salon and in the apprenticeship school, showing they could be measured with good analytical sensitivity after personal sampling lasting 3 hours and more.

#### - Air sampling

Personal exposure measurements were done during a complete 5 to 8 hours work shift, the apprentices carrying the sampler installed in a rucksack; sampling heads were near the breathing zone. The Harvard Chempass sampler [28] was used during the first year measurements (winter and summer 2004; 8 measurements), when only two compounds were measured (persulfate and ammonia), a portable pump BGI (model 400) providing an air flow of 1.8 l/min in each head. For practical reasons (weight of the BGI pump), Gillian pumps were used the second year (winter and summer 2005), with a 1 l/min airflow for the three compounds. The total weight of the sampling devices was thus reduced, allowing to measure in parallel the three selected compounds. Data drawn with the two pumps were lumped together.

Environmental sampling was done by Gillian pumps with a 1 l/min airflow for the three compounds over the whole study period. Environmental sampling equipments were deposited at the beginning of the apprentice's work shift, in parallel to the personal exposure measurements. All samplers (environmental and personal exposure) were stopped during the lunch break in case it took place out of the salon.

#### - Chemical analyses methods

For analyses of hydrogen peroxide, quartz fibre filters were impregnated with 210 μl oxisulfate of titanium and quantification was done by colorimetry [29]. Persulfates were sampled on 37 mm Teflon filters of 1 μm pore size and analyses were performed according to the BIA method [30]. Persulfates were determined with a ionchromatograph, Dionex model DX 600, LiChrospher column 100 RP-18 (5 μm, flow of 1 ml/min) with the following eluents: tetrabutylammoniumhydroxide 0.005 mole/l, acid boric 0.0075 mole/l and acetonitril 22%. Ammonia was collected on quartz filter impregnated by H_2_SO_4_, 1.5 mol/l. The analyses used ionchromatography, Dionex, model DX 600, Dionex CS12 column with, as eluents: carbonate/bicarbonate (2.7 milliMole of sodium carbonate, 0.3 milliMole of sodium bicarbonate) at a flow of 1 ml/min [31]. Detection limits were 0.006 mg/m^3^, 0.0005 mg/m^3 ^and 0.003 mg/m^3 ^for H_2_O_2_, H_2_S_2_O_8_and NH_3 _respectively. The INRS laboratory where the chemical analyses were performed follows QA/QC procedures controlled by a dedicated national institution (Comité Français d'Accréditation, COFRAC).

### Sample size and statistical analysis

Three hundred students completed the questionnaires in the apprenticeship school. An important drop occurred between this sample size and the number of those who contributed to the exposure measurements. Among the 35 subjects who volunteered, 12 employers declined participation. Three cases were discarded: two because the hairdressing salon was too far, and one because the apprentice had to quit school for health reasons. In addition, three salons could not be visited in the hot season because of schedule constraints, leaving 28 ('cold season') and 25 ('hot season') measurements. Because the hairdressers had limited availability to answer the telephone calls, on average, 12 calls were necessary to obtain a final agreement and set a date for the measurements.

To compare exposures and air concentrations values we used the non parametric paired rank test because distributions were not Gaussian nor log-normal; average workplace concentrations were computed (with 2 to 3 data according to locations) after a check of their similarity. All values lower than the detection limit were set to half the detection limit; 2 (5.1%) of the data were in this case for H_2_O_2 _personal exposure values, and none of the NH_3 _and persulfates personal exposures values (corresponding figures were respectively 15 (9.6%), 0 (0%) and 2 (1.3%) for H_2_O_2_, NH_3 _and persulfates workplace concentrations). To study the influence of salon characteristics on exposures, we used multivariate general linear models where the salon volume, presence of ventilation devices or natural ventilation during the sampling period (i.e. door or windows opened outside) and season were the explanatory variables. Questionnaires data were input with Epidata. Statistical analysis was carried out using the SASstatistical package and Statgraphics.

## Results

### Study population and work environment

Apprentices were almost all females (table 1). Since most apprentices were in salons for both men and women (one was in men-only and 4 were in women-only facilities), the following results will not differentiate salons according to gender customers. Table 2, based on questionnaires data, shows the distribution of declared time spent at work according to the training level with, as expected, longer shifts among second and third graders.

**Table 1:** Description of the study population.

	All apprentices (n = 300)	Exposure volunteers (n = 28)
Gender		
Female	293 (97,7)	27 (96,4)
Male	7 (2,3)	1 (3,6)
Training level		
First year	73 (24.3)	4 (14,3)
Second year	141 (47.0)	17 (60,7)
Third year	86 (28.7)	7 (25.0)

**Table 2:** Duration of daily shift according to apprenticeship training level.

		[6–8 h [(%)	[8–10 h [(%)	[10–12 h] (%)
	First year (n = 73)	37 (50.7)	30 (41.1)	5 (6.9)
CAP	Second year (n = 141)	42 (29.8)	87 (61.7)	12 (8.5)
	Third year (n = 86)	23 (26.8)	53(61.6)	10 (11.6)

There were 3 types of spatial settings: 144 apprentices declared their salon was organized with specific areas (haircut and brushing spot; shampoo spot; technical spot where creams and solutions were prepared) within a single volume; 51 reported different rooms for these activities; and 102 were in a homogeneous single space with no area differentiation. One third of customer spaces and two thirds of technical spaces, when existing, had no ventilation device (fan, air conditioning or other type of venting, like ceiling fan) (table 3). The size of the salons where measurements were carried out spanned from 52.5 to 288 m^3 ^(average 143.68 [sd:62.4] m^3^).

**Table 3:** Proportion of salons with no ventilation nor direct opening outside, by salon area.

	No ventilation	No door opening outside	No window opening outside
Customers space	32.8 (259)^a^	4.4 (298)^a^	53.2 (297)^a^
Technical space	66.1 (218)^a^	51.9 (262)^a^	63.3 (253)^a^

a: number of questionnaires completed.

The tasks that are associated with manipulation of chemicals are described in table 4 which exhibits how frequent these tasks are accomplished during a typical day, as reported by questionnaires. Data are split according to training level; corresponding figures for apprentices who volunteered to participate to the exposure measurements are in the last column. Tasks frequencies varied markedly according to training year (all p values after Kruskal-Wallis tests were smaller than 0.03), except for rinsing hair bleaches (p = 0.42). Daily numbers of services using chemicals for permanent waves and of hair colour rinsing are greater, on average, among second year students, while preparing and applying hair colour and hair bleaches is more frequent among third graders.

**Table 4:** Daily frequency of activities associated with manipulation of chemicals (in % of respondents).

		First year	Second year	Third year	Volunteers
		(n = 73)^a^	(n = 141)^a^	(n = 86)^a^	(n = 28)^a^
		(max = 68)^b^	(max = 137)^b^	(max = 86)^b^	(max = 26)^b^
		(min = 59)^c^	(min = 131)^c^	(min = 82)^c^	(min = 24)^c^
Application of permanent waving	0 per day	27.9	18.2	14.5	23.1
	[1 – 2]	45.9	38.6	49.4	46.2
	[3 – 5]	24.6	34.9	32.5	26.9
	[6 – 15]	1.6	8.3	3.6	3.8
Rinsing permanent waving	0 per day	3.2	6.7	10.6	4.0
	[1 – 2]	62.9	38.5	47.1	52.0
	[3 – 5]	30.7	45.9	37.6	40.0
	[6 – 15]	3.2	8.9	4.7	4.0
Applying neutralizing agent	0 per day	9.8	8.3	10.5	4.2
	[1 – 2]	63.9	39.8	46.5	54.1
	[3 – 5]	24.6	42.9	34.9	37.5
	[6 – 15]	1.7	9.0	8.1	4.2
Preparing hair colour	0 per day	17.2	9.8	1.2	4.0
	[1 – 4]	32.8	22.5	25.9	4.0
	[5 – 9]	35.9	37.6	40.0	64.0
	[10 – 25]	14.1	30.1	32.9	28.0
Applying hair colour	0 per day	10.3	3.6	0	3.8
	[1 – 4]	48.5	33.6	26.5	30.8
	[5 – 9]	32.4	36.5	47.0	38.5
	[10 – 22]	8.8	26.3	26.5	26.9
Rinsing hair colour	0 per day	0	1.5	1.2	0
	[1 – 4]	32.8	18.7	20.2	15.4
	[5 – 9]	47.8	34.3	53.6	50.0
	[10 – 25]	19.4	45.5	25.0	34.6
Preparing hair bleaches	0 per day	45.9	23.9	16.9	12.0
	[1 – 2]	22.9	34.6	40.9	44.0
	[3 – 6]	14.8	24.6	24.1	28.0
	[7 – 20]	16.4	16.9	18.1	16.0
Applying hair bleaches	0 per day	64.4	34.4	23.2	32.0
	[1 – 2]	22.0	40.5	39.0	44.0
	[3 – 6]	6.8	19.8	25.6	20.0
	[7 – 20]	6.8	5.3	12.2	4.0
Rinsing hair bleaches	0 per day	31.7	15.3	14.3	24.0
	[1 – 2]	30.2	43.5	42.9	48.0
	[3 – 6]	22.2	30.5	28.6	24.0
	[7 – 20]	15.9	10.7	14.3	4.0

a: numbers apprentices by level; b: maximum number of respondents for the set of questions; c: minimum number of respondents for the set of questions.

### Exposure measurements

As shown in table 1, apprentices who participated in the exposure study were mostly in their second year of training. Table 5 presents personal exposure levels along with concentrations of the 3 measured chemicals in different locations of the hairdressing salons. Personal exposures to H_2_O_2 _(n = 39; mean 0.051 [sd: 0.042] mg/m^3^) and NH_3 _(n = 52; 0.900 [sd: 0.762] mg/m^3^) are greater than average workplace concentrations (0.037 [sd: 0.031] mg/m^3 ^and 0.677 [sd: 0.425] mg/m^3^, respectively) (winter and summer data altogether, because they did not differ: all paired test p values being equal to or greater than 0.11). The opposite holds true for H_2_S_2_O_8 _(n = 51; 0.016 [sd: 0.021] mg/m^3 ^and 0.019 [sd: 0.018] mg/m^3^, respectively). We found no significant association between salon volumes or ventilation means and personal exposures, irrespective of the measured compound (all p values greater than 0.13).

**Table 5:** Personal exposures and workplace concentrations for ammonia, hydrogen peroxide and persulfates in different salon areas.

	**H_2_O_2_**	**NH_3_**	**H_2_S_2_O_8_**
Personal exposure	(n = 39)^a^	(n = 53)	(n = 52)
	0.05^b ^(0.04)^c^	0.90 (0.76)	0.02 (0.02)
	[0.003*-0.18]^d^	[0.02–4.49]	[0.001–0.12]
Technical area in the customer salon (permanenting, dyeing, bleaching with the customers)	(n = 53)	(n = 52)	(n = 52)
	0.04 (0.03)	0.72 (0.49)	0.02 (0,02)
	[0.003*-0.18]	[0.13–3.03]	[0.00025*-0.1]
Shampoo area	(n = 51)	(n = 51)	(n = 51)
	0.03 (0.03)	0.65 (0.40)	0.02 (0,02)
	[0.003*-0.13]	[0.09–2.39]	[0.00025*-0.13]
Technical room (mixing the chemicals for permanenting, dyeing and bleaching)	(n = 52)	(n = 52)	(n = 52)
	0.04 (0.05)	0.66 (0.45)	0.02 (0,02)
	[0.003*-0.34]	[0.13–2.64]	[0.002–0.08]
Average of ambient air concentrations in each salon	(n = 53)	(n = 52)	(n = 52)
	0.04 (0.03)	0.68 (0.42)	0.02 (0,02)
	[0.003*-0.15]	[0.13–2.69]	[0.002–0.08]

a: Each cell comprises the number of samples; b: the arithmetic mean; c: standard deviate; d: [min-max] (in mg/m^3^)

* These min. figures were set to half the detection limit (see chapter materials and methods).

## Discussion

Although a larger sample for the exposure study population was expected, achieving this objective was difficult for many reasons. First, occupational health is not regarded as of serious concern in this population of teenagers. Appreciation of respiratory diseases prevention was low among apprentices, an observation also made by Wong *et al *among hairdressing students [32]. Second, many apprentices were reluctant to accept participation because they anticipated refusal or feared rebuttal by their boss. Third, the constraints posed to volunteers and to the hairdressing managers cannot be underestimated, including with respect to the salon costumers. For these reasons, it was not possible to sample participating salons among different categories of facilities (small, large; within commercial malls or in solo), as we had planned. Our experience, however, is that the types of facilities and indoor environments of hairdressing salons do not vary greatly in the Lorraine region. Hence, while this is not a random sample, exposure data can be viewed as indicative of typical values currently found in such facilities.

Questionnaires were designed to obtain descriptive information on apprentices' work conditions in hairdressing salons. In order to minimize recall bias, individual tasks we explored for a typical weekday, to be chosen within the ongoing week; it was frequently the previous work day. We did not attempt to study the busiest day, as Labreche *et al *did [33]; however, the number of tasks that are accomplished in our study is in the same order as those they reported. Hair colouring is the task most often practiced among the chemical services, more than permanente waving and hair bleaching. According to Kersemaekers, however, hairdressers tend to overestimate their work load; also, they do not know accurately the type of ventilation in their facility [34].

While the observed greater personal exposure values, as opposed to H_2_O_2 _and NH_3 _workplace concentrations, were expected, in accord with the 'personal cloud' concept, we found opposite results for H_2_S_2_O_8_. This may be explained by two reasons. Apprentices were less involved in bleaching than in hair dyeing and permanenting (table 4) because, being a more technical task (both for bleaching mixture preparation and its application), it is more often accomplished by senior hairdressers. Further, workplace air samplers were located in a place deemed representative of the area to be monitored. Hence, in this case of activity imbalance within the salon work team, fixed samplers were liable to be more influenced by the tasks executed by senior hairdressers than by the study apprentice. This was not the case for compounds associated with hair dyes and permanenting, because these tasks were frequently accomplished by apprentices.

Comparison of exposure and concentrations results across the literature should be done with caution because time and space sampling procedures, measurement techniques and methods of analyses are not always comparable. Other differences across studies may be due to exposure sampling designs and stem from the fact that working conditions may vary across study locations. In the study by Hollund *et al*., for example, measurements were done during the phase of preparation of the technique, with a view to assess peak concentrations, also taking into account certain parameters of ventilation [23]. We found that more than half of technical areas did not have ventilation systems nor did they open outside (through a door or a window). For the American Society of Heating Refrigerating and Air Conditioning Engineers, ventilation rates in beauty salons should ensure values of 20 cubic feet/minute/person, and 7.5 cfm/person for barber shops (minimum ventilation rates breathing zone) [35].

Our results for personal exposures to ammonia show maximum values similar to those described by Hollund *et al *for dyeing and waving (4.4 mg/m^3^, versus 4.5 mg/m^3 ^during our 'cold period') but Hollund *et al *found levels up to 10 mg/m^3 ^during bleaching, in absence of exhaust ventilation. Also, while average exposure values were greater than in our study, workplace ambient concentrations in both studies are close [23]. The study by Leino *et al *aimed at measuring peak concentrations during the process of dyeing, permanenting and bleaching; it shows values 1.3 times smaller than our maximum personal exposure measurements [27]. Although the 2 minutes spot exposure measurements found by Van der Wal are very high compared to our values, their ambient air measurements are similar to ours [22]. It should be noted that all these results were below the French and Belgium TLVs for ammonia (7 and 14 mg/m^3^, respectively) [36,37], the NIOSH TWA-REL for ammonia (18 mg/m^3^) and the OSHA TWA-PEL (35 mg/m^3^) [38].

Average personal exposures to H_2_O_2 _in our study are 1.4 times lower than those found by Van der Wal *et al *during the process of colour rinsing, and 2.8 times lower during bleaching (exposures were 2 minutes spot measurements in the Van der Wal *et al *study). This study also describes greater exposure figures during waiving (0.14–1.4 mg/m^3^, during 2 minutes spot exposure measurements) [22]. Average air concentrations during a shift, however, are between 2 times lower than our results, respectively in winter and summer [22]. The German BIA designed a hairdressing salon and performed a quasi-experimental study. The sampling site close to the armchair showed a maximum H_2_O_2 _value 1.6 times higher than ours in customer salons. Also, the maximum concentrations measured in the hairdressing laboratory were 2.4 times greater than our maximum value in technical areas, during the winter period measurements [30]. French and Belgium TLVs, NIOSH REL TWA, OSHA PEL TWA for H_2_O_2 _are 1.5 and 1.4 mg/m^3^, respectively [36-38].

Our maxima personal exposure results during summer are 3.8 times greater than the peak values found by Leino *et al *during mixing of bleaching powder. Our average workplace concentrations are also respectively 6.6 and 21.1 times higher than those found in the small and large salons they studied [27]. These results are worrisome since persulfate salts are important asthma causal agents [3,16,18,19,27]. On the other hand, results from the BIA study exceed our maxima workplace concentrations [30]. It should be noted, however, that the former stem from short periods measurements, focused in particular when mixtures of hairdressing products were made, while our data correspond to shift averages over all daily activities. To our knowledge, there is no TLV for persulfates as a whole; a persulfate ammonium TLV of 0.1 mg/m^3 ^has been set by the US ACGIH and in Belgium [37,39].

## Conclusion

Ventilation is not frequent in the study hairdressing salons. Manipulation of bleaching products, among other chemical substances used in the hairdressing process, yields exposure to airways irritants. Although lower than current occupational limit values, these exposures might be of health significance. These findings suggest that further occupational hygiene progresses may result from a combination of efforts dealing with safer products presentation, improvements in facility ventilation equipment, and information of hairdresser, including young apprentices during their training, as to potential hazards in the workplace.

## Abbreviations

NH_3 _= Ammonia

H_2_O_2 _= Hydrogen peroxide

H_2_S_2_O_8 _= Persulfates

## Competing interests

The author(s) declare that they have no competing interests.

## Authors' contributions

EMG coordinated the study, designed the questionnaire, conducted field investigations and performed the statistical analysis. She is the main author of the manuscript. VO performed all chemical analyses. LM contributed to the statistical analysis. CP was an advisor for the study design and contributed to writing the paper. DZN directed the study and supervised writing of the manuscript. All authors read and approved the final manuscript.

## References

[B1] Ameille J, Pauli G, Calastreng-Crinquand A, Vervloët D, Iwatsubo Y, Popin E, Bayeux-Dunglas MC, Kopferschmitt-Kubler MC (2003). Reported incidence of occupational asthma in France, 1996–99: the ONAP programme. Occup Environ Med.

[B2] Malo JL, Chan-Yeung M (2001). Occupational asthma. J Allergy Clin Immunol.

[B3] Moscato G, Pignatti P, Yacoub MR, Romano C, Spezia S, Perfetti L (2005). Occupational asthma and occupational rhinitis in hairdressers. Chest.

[B4] Blainey AD, Ollier S, Cundell D, Smith RE, Davies RJ (1986). Occupational asthma in a hairdressing salon. Thorax.

[B5] Borelli S, Wüthrich B (1999). Immediate and delayed hypersensitivity to ammonium persulfate. Allergy.

[B6] Parra FM, Igea JM, Quirce S, Ferrando MC, Martin JA, Losada E (1992). Occupational asthma in a hairdresser caused by persulphate salts. Allergy.

[B7] Schwartz HJ, Arnold JL, Strohl KP (1990). Occupational allergie rhinitis in the hair care industry: reactions to permanent wave solutions. J Occup Med.

[B8] NIOSH (1976). Health hazard determination report: Radiant Lady Beauty Salon, Inc., Denver, CO. Cincinnati, OH: US Department of Health and Human Services, Public Health Service, National Institute for Occupational Safety and Health, NIOSH Report No75-128-262.

[B9] Heacock HJ, Rivers JK (1986). Occupational diseases of hairdressers. Can J Public Health.

[B10] Pepys J, Hutchcroft BJ, Breslin AB (1976). Asthma due to inhaled agents-persulphate salts and henna in hairdressers. Clin Allergy.

[B11] Slater T, Bradshaw L, Fishwick D, Cheng S, Kimbell-Dunn M, Erkinjuntti-Pekkanen R, Douwes J, Pearce N (2000). Occupational respiratory symptoms in New Zealand hairdressers. Occup Med.

[B12] Leino T, Tammilehto L, Luukkonen R, Nordman H (1997). Self reported respiratory symptoms and diseases among hairdressers. Occup Environ Med.

[B13] Akpinar-Elci M, Cimrin AH, Elci OC (2002). Prevalence and Risk Factors of Occupational Asthma Among Hairdressers in Turkey. J Occup Environ Med.

[B14] Leino T, Tammilehto L, Paakkulainen H, Orjala H, Nordman H (1997). Occurrence of Asthma and Chronic Bronchitis Among Female Hairdressers. J Occup Environ Med.

[B15] Schwaiblmair M, Vogelmeier C, Fruhmann G (1997). Occupational asthma in hairdressers: results of inhalation tests with bleaching powder. Int Arch Occup Environ Health.

[B16] Moscato G, Galdi E (2006). Asthma and hairdressers. Curr Opin Allergy Clin Immunol.

[B17] Hollund BE, Moen BE, Lygre SH, Florvaag E, Omenaas E (2001). Prevalence of airway symptoms among hairdressers in Bergen, Norway. Occup Environ Med.

[B18] Munoz X, Cruz MJ, Orriols R, Bravo C, Espuga M, Morell F (2001). Occupational asthma due to persulfate salts : diagnosis and follow-up. Chest.

[B19] Munoz X, Cruz MJ, Orriols R, Torres F, Espuga M, Morell F (2004). Validation of specific inhalation challenge for the diagnosis of occupational asthma due to persulphate salts. Occup EnvironMed.

[B20] Gagliardi L, Ambroso M, Mavro J, Furno F, Discalzi G (1992). Exposure to paraphenylendiamine in hairdressing parlours. Int J Cosmet Sci.

[B21] Van Muiswinkel JV, Kromhout H, Onos T, Kersemaekers W (1997). Monitoring and modelling of exposure to ethanol in hairdressing salons. Ann occup Hyg.

[B22] Van der Wal JF, Hoogeveen AW, Moons AMM, Wouda P (1997). Investigation on the exposure of hairdressers to chemical agents. Environ Int.

[B23] Hollund BE, Moen BE (1998). Chemical Exposure in Hairdressers Salons: Effect of Local Exhaust Ventilation. Ann occup Hyg.

[B24] Arcangeli G, Baldasseroni A, Palmi S, Bianchi A (1999). [Reversible pulmonary response to irritating substances: study on a population of apprentice hairdressers.]. Prevenzione oggi.

[B25] Gülmez Ý, Çetinkaya F, Oymak FS, Demir R, Özesmi M (1998). Occupational asthma among hairdresser's apprentices [abstracts]. Europ Respir J Suppl.

[B26] Iwatsubo Y, Matrat M, Brochard P, Ameille J, Choudat D, Conso F, Coulondre D, Garnier R, Hubert C, Lauzier F, Romano MC, Pairon JC (2003). Healthy worker effect and changes in respiratory symptoms and lung function in hairdressing apprentices. Occup Environ Med.

[B27] Leino T, Kähkönen E, Saarinen L, Henriks-Eckerman M-L, Paakkulainen H (1999). Working Conditions and Health in Hairdressing Salons. Appl Occup Environ Hyg.

[B28] Demokritou P, Kavouras IG, Ferguson ST, Koutrakis P (2001). Developpement and laboratory performance evaluation of a personal multipollutant sampler for simultaneous measurements of particulate and gaseaous pollutants. Aerosol Sci Technol.

[B29] Métropol. Fiche 047 : Peroxyde d'hydrogène. http://www.inrs.fr/.

[B30] Ermittlung der Atemwegsbelastungen durch luftfremde Stoffe an Friseurarbeitsplätzen. Abschlussbericht. BIA, Bericht Nr.: 36/199920117. Sankt Augustin;.

[B31] Métropol. Fiche 013 : Ammoniac et sels d'ammonium. http://www.inrs.fr/.

[B32] Wong RH, Chien HL, Luh DL, Lin WH, Wang YC, Cho CY (2005). Correlation Between Chemical-Safety Knowledge and Personal Attitudes Among Taiwanese Hairdressing Students. Am J Ind Med.

[B33] Labreche F, Forest J, Trottier M, Lalonde M, Simard R (2003). Characterization of chemical exposures in hairdressing salons. Appl Occup Environ Hyg.

[B34] Kersemaekers WM, Verheijen N, Kromhout H, Roeleveld N, Zielhuis GA (1998). Assessment of exposure to solvents among hairdressers: reliability of a classification scheme and questionnaire. Occup Environ Med.

[B35] (2004). American Society of Heating, Refrigerating and Air-Conditioning Engineers (ASHRAE). ANSI/ASHRAE Standard 621-2004 – Ventilation for Acceptable Indoor Air Quality (ANSI Approved).

[B36] Institut National de Recherche et de Sécurité INRS (2005). Valeurs limites d'exposition professionnelle aux agents chimiques en France ND 2098 Note Documentaire Mise à jour février 2005 France.

[B37] Arrêté royal du 11 mars 2002 relatif à la protection de la santé et de la sécurité des travailleurs contre les risques liés aux agents chimiques sur le lieu de travail (MB 14.3.2002 Ed. 2;erratum M.B. 26.6.2002, Ed. 2;erratum M.B. 4.12.2002). Annexe 1 Valeurs limites d'exposition professionnelle.

[B38] (2005). Department of health and human services. Centers for Disease Control and Prevention. National Institute for Occupational Safety and Health. Niosh pocket guide to chemical hazards.

[B39] American Conference of Governmental Industrial Hygienists ACGIH (2005). Annual Reports of the Committees on TLVs® and BEIs® for Year 2005.

